# Isolation and Characterization of Tumor Cells from the Ascites of Ovarian Cancer Patients: Molecular Phenotype of Chemoresistant Ovarian Tumors

**DOI:** 10.1371/journal.pone.0046858

**Published:** 2012-10-08

**Authors:** Ardian Latifi, Rodney B. Luwor, Maree Bilandzic, Simon Nazaretian, Kaye Stenvers, Jan Pyman, Hongjian Zhu, Erik W. Thompson, Michael A. Quinn, Jock K. Findlay, Nuzhat Ahmed

**Affiliations:** 1 Women’s Cancer Research Centre, Royal Women’s Hospital, Victoria, Australia; 2 Department of Surgery, University of Melbourne, St Vincent Hospital, Victoria, Australia; 3 Department of Surgery, University of Melbourne, Royal Melbourne Hospital, Victoria, Australia; 4 Prince Henry’s Institute for Medical Research, Victoria, Australia; 5 Department of Anatomical Pathology, Royal Women’s Hospital, Victoria, Australia; 6 St Vincent Institute, Victoria, Australia; 7 Department of Obstetrics and Gynaecology, University of Melbourne, Victoria, Australia; Baylor College of Medicine, United States of America

## Abstract

Tumor cells in ascites are a major source of disease recurrence in ovarian cancer patients. In an attempt to identify and profile the population of ascites cells obtained from ovarian cancer patients, a novel method was developed to separate adherent (AD) and non-adherent (NAD) cells in culture. Twenty-five patients were recruited to this study; 11 chemonaive (CN) and 14 chemoresistant (CR). AD cells from both CN and CR patients exhibited mesenchymal morphology with an antigen profile of mesenchymal stem cells and fibroblasts. Conversely, NAD cells had an epithelial morphology with enhanced expression of cancer antigen 125 (CA125), epithelial cell adhesion molecule (EpCAM) and cytokeratin 7. NAD cells developed infiltrating tumors and ascites within 12–14 weeks after intraperitoneal (i.p.) injections into nude mice, whereas AD cells remained non-tumorigenic for up to 20 weeks. Subsequent comparison of selective epithelial, mesenchymal and cancer stem cell (CSC) markers between AD and NAD populations of CN and CR patients demonstrated an enhanced trend in mRNA expression of E-cadherin, EpCAM, STAT3 and Oct4 in the NAD population of CR patients. A similar trend of enhanced mRNA expression of CD44, MMP9 and Oct4 was observed in the AD population of CR patients. Hence, using a novel purification method we demonstrate for the first time a distinct separation of ascites cells into epithelial tumorigenic and mesenchymal non-tumorigenic populations. We also demonstrate that cells from the ascites of CR patients are predominantly epithelial and show a trend towards increased mRNA expression of genes associated with CSCs, compared to cells isolated from the ascites of CN patients. As the tumor cells in the ascites of ovarian cancer patients play a dominant role in disease recurrence, a thorough understanding of the biology of the ascites microenvironment from CR and CN patients is essential for effective therapeutic interventions.

## Introduction

In 2009, the American Association for Cancer Research reported ovarian cancer as the gynecological malignancy with the highest case-to-mortality ratio [1]. This high mortality rate results from the diagnosis at an advanced-stage when the cancer has spread into the peritoneal cavity and metastasized to vital organs. Ovarian cancer metastasis occurs either directly from the cortical inclusion cysts of the ovaries or from the fimbrial end of the fallopian tube [2], and spreads by direct extension to adjacent organs (for example extraovarian pelvic organs, colon, bladder, liver, etc), or by the attachment of exfoliated ovarian cancer cells which survive as cellular aggregates or spheroids. Spheroids are carried by the peritoneal tumor fluid (ascites) to surrounding organs in the peritoneal cavity. Extensive seeding of these spheroids on the uterus, sigmoid colon and omentum is frequently encountered in advanced-stage and recurrent disease [3].

**Table 1:** Description of patients included in the study.

Sample number	Stage	Age	Treatment	Status	Years survived
1	IIIC	55	chemonaive	alive	1 year 3 months
2	unknown	65	MORb trial 1, carboplatin and paclitaxel 6 cycles	alive	2 years 1 month
3	IIIc	60	chemonaive	alive	3 months
4	IIIc	48	chemonaive	deceased	1 year 2 months
5	unknown	80	carboplatin and paclitaxel combination 6 cycles, doxorubicin 1cyle	deceased	1 year 6 months
6	IIc	54	chemonaive	alive	3 months
7	IIIc	90	chemonaive	deceased	1 month
8	IIIc	68	cyclophosphamide 1 cycle, carboplatin and paclitaxel and carboplatin	alive	3 years 10 months
9		60	chemonaive	alive	6 months
10	IIIc	68	cyclophosphamide 1 cycle, carboplatin and paclitaxel and carboplatin6 cycles	alive	3 years 10 months
11	IIIc	54	paclitaxel 2 cycles, carboplatin and paclitaxel 3 cycles, doxorubicin4 cycles AMG 2 cycles, cyclophosphamide 3 cycles	deceased	1 year 8 months
12	IIIc	79	cyclophosphamide 4 cycles, carboplatin2 cycles, docetaxel 1 cycle	deceased	9 months
13	IIIc	51	docetaxel 3 cycles, unspecified regime 1 cycle, carboplatin andpaclitaxel 6 cycles, doxorubicin 3 cycles, carboplatin 1 cycle,gemcitabine and carboplatin 6 cycles, docetaxel 3 cycles, topotecan2 cycles, paclitaxel 4 cycles	deceased	2 years 11 months
14	IIIb	65	carboplatin and paclitaxel combination 6 cycles	deceased	3 years
15	IIIc	65	chemonaive	alive	1 year 11 months
16	IIIc	56	chemonaive	alive	2 years 3 months
17	unknown	54	topotecan 1 cycle, doxorubicin 4 cycles, carboplatin and paclitaxelcombination 6 cycles	alive	4 years 5 months
18	IV	53	chemonaive	alive	2 years 4 months
19	unknown	72	chemonaive	alive	9 months
20	IIIc	39	MORb trial 6 cycles, doxorubicin 9 cycles, gemcitabine andcarboplatin 2 cycles	deceased	3 years 7 months
21	Ic	33	carboplatin & paclitaxel combination 6 cycles	deceased	2 years 9 months
22	IIIc	66	chemonaive	alive	11 months

Current treatment strategies for advanced-stage ovarian cancer patients result in initial remission in up to 80% of patients [4]. However, after a short remission period (usually 6–22 months) recurrence occurs in almost all patients [4]. This is largely due to the ability of tumor cells to evade chemotherapy-associated cytotoxicity through acquired chemoresistance. Recently, chemoresistance has also been associated with the acquisition of epithelial to mesenchymal transition (EMT) in cancer cells [5]–[6]. Classically, EMT enables stationary epithelial cells to become motile and invasive in order to spread and recolonize into surrounding tissues [7]. These features of EMT have been shown to correlate with a CSC-like phenotype [8]–[9], corroborated recently in clinical cases by the mesenchymal and ‘tumor initiating’ phenotypes of the residual tumor cells in breast cancer patients surviving conventional therapy [10]. The phenotype of CSC has been shown to be dynamically regulated by the tumor microenvironment [11], and the key feature required for micro and macro-metastatic colonization involves not only EMT but also mesenchymal to epithelial transition (MET) [12]–[13]. The continuum of EMT and MET has been described as epithelial mesenchymal plasticity (EMP) [11]. Such metastatic colonization defines the ability of EMP transformed disseminated tumor cells to self-renew and differentiate, the defining cellular traits of CSCs [14].

**Table 2:** Description of the patients recruited for the animal study.

Sample number	Stage	Age	Treatment	Status	Years survived
1	IIIc	39	MORb trial 6 cycles, doxorubicin 9 cycles, gemcitabine and carboplatin2 cycles	deceased	3 years 7 months
2	IIIc	68	cyclophosphamide 1, carboplatin and paclitaxel Carboplatin 6 cycles	alive	3 years 10 months
3	IIIc	57	Icon7 trial 6 cycles, carboplatin 5 cycles, cyclophosphamide 1 cycle	deceased	3 years 11 months

Although the presence of ascites has been associated with poor prognosis, the origin and phenotype of cancer cells in ascites, and its association with chemoresistance and recurrence is poorly understood. Microscopic inspection of ascites has previously revealed a complex heterogeneous image consisting of single cells and spheroids [15]. Non-cancer cells within the ascites include inflammatory cells, cancer-associated fibroblasts, immature myeloid cells and activated mesothelial cells, all of which influence tumor cell behavior and response to chemotherapy [16]. Also contributing to the heterogeneity of the ascites is a population of CSCs that can resist chemotherapy and give rise to a hierarchy of proliferating tumor cells with progressive differentiating potential [17], [18]. These CSCs, when purified by sorting and xenografted into nude mice, have been shown to generate a significantly greater tumor burden compared to unsorted tumor cells [19], suggesting the greater tumorigenic potential of CSCs.

**Figure 1:**
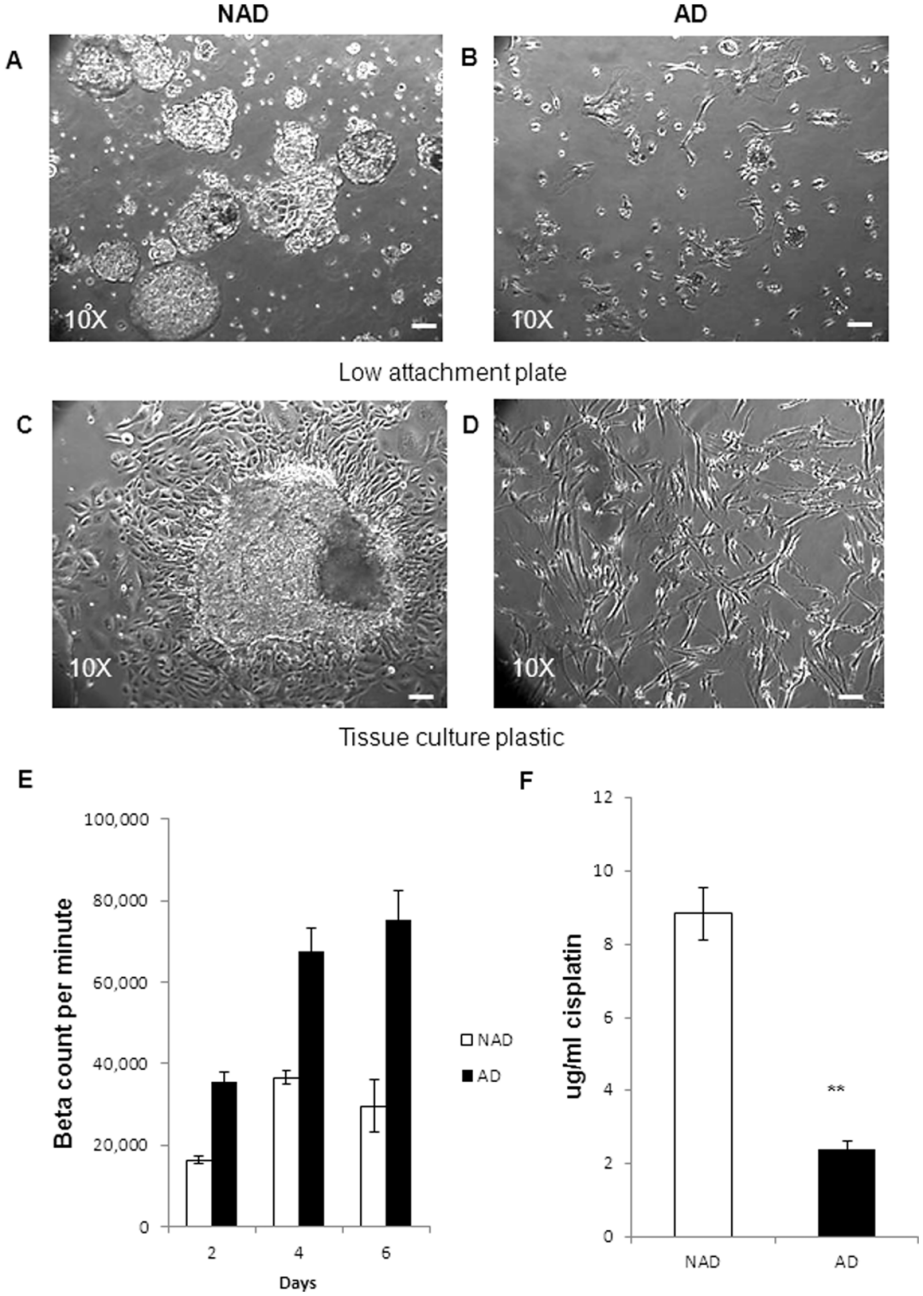
Morphological features, proliferation and cisplatin sensitivity of NAD and AD cells. (**A**) NAD spheroids and (**B**) AD cells were seeded on low attachment plates immediately after collection. Morphological features of (**C**) NAD spheroid and (**D**) AD cells on tissue culture plastic after 24 h following seeding. Images were assessed by phase contrast microscopy. Magnification was 100×, scale bar = 50 µm. The images are representative of (n = 25) samples. (**E**) [^3^H]-thymidine uptake in AD cells and in cells dispersed from spheroids was performed as described in Methods and Materials. The graph is a representation of one ascites sample performed in triplicate. (**F**) Effect of cisplatin on the proliferation {[^3^H]-thymidine uptake} of NAD and AD cells obtained from the ascites of ovarian cancer patients. The graph is a representation of three independent experiments, performed on three independent NAD and AD samples in triplicate. Significantly different between AD versus NAD cells, **p<0.01.

We hypothesize that recurrence in ovarian cancer patients is largely dictated by the extent to which the tumor and associated stromal cells in the peritoneal cavity survive chemotherapy, and that a comparative mRNA study of the cells isolated from the ascites of CN versus CR ovarian cancer patients may provide an important missing link to understanding recurrent disease. The overall aim of this study was to investigate the differential mRNA profile of ascites cells from CN and CR patients in order to identify the gene products that may contribute to the survival and spread of residual cancer cells following chemotherapy. We also aimed to understand the metastasizing propensity of the tumor cells in the ascites of ovarian cancer patients. To achieve this, we developed a novel purification method to isolate distinct populations of cells from the ascites of ovarian cancer patients. Using this simple technique ascites cells were separated in two distinct populations of cells with well-defined epithelial and mesenchymal phenotypes, respectively. Cells isolated from CR ascites had a more epithelial phenotype and showed a trend towards enhanced expression of genes associated with CSC when compared to cells isolated from the ascites of CN patients. These results suggest that the ascites tumor microenvironment may differ in patients before and after chemotherapy and further may play a role in the relapse of ovarian cancer patients post-chemotherapy.

**Figure 2:**
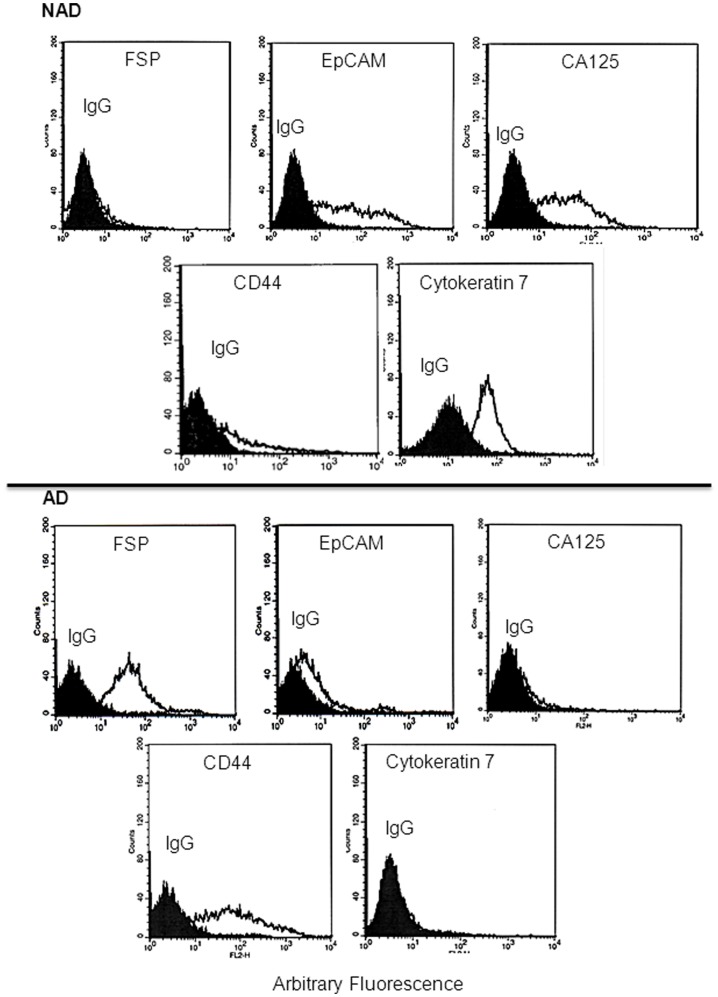
Expression of surface markers on NAD and AD cells by flow cytometry. Purified cells from the ascites of CN (n = 11) and CR (n = 14) ovarian cancer patients were incubated with either control IgG or relevant primary antibodies against the respective antigens followed by secondary phycoerythrin conjugated antibody. Results are representative of (n = 25) independent samples. The filled histogram in each figure represents control IgG, black lines indicate protein expression in respective cells.

## Materials and Methods

### Antibodies and Reagents

Monoclonal and polyclonal antibodies against CA125, fibroblast surface protein (FSP) and CD44 were obtained from Merck Millipore (MA, USA). Monoclonal antibodies against cytokeratin 7 (cyt 7) and N-cadherin were obtained from Zymed Laboratories (San Francisco, USA). Polyclonal antibodies against E-cadherin, vimentin and EpCAM were obtained from Cell Signalling Technology (Beverly, MA, USA). Polyclonal antibodies against CD73, CD105, CD90, and CD34 were obtained from Sapphire Bioscience (NSW, Australia).

**Figure 3:**
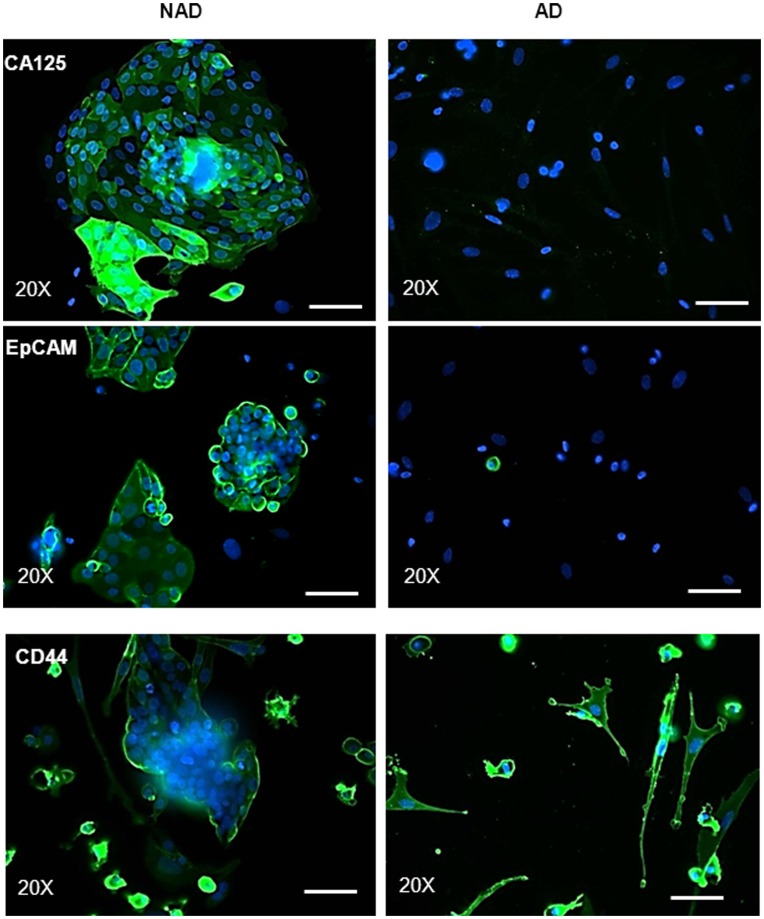
Expression and immunolocalization of CA125 and CSC markers by immunofluorescence. Purified NAD and AD cells were evaluated by immunofluorescence using mouse monoclonal antibody (green) as described in the Methods and Materials. Cellular staining was visualized using the secondary Alexa 488 (green) fluorescent labeled antibody, and nuclei were detected by DAPI (blue) staining. Images are representative of three independent samples. Magnification was 200×; scale bar = 50 µm.

**Figure 4:**
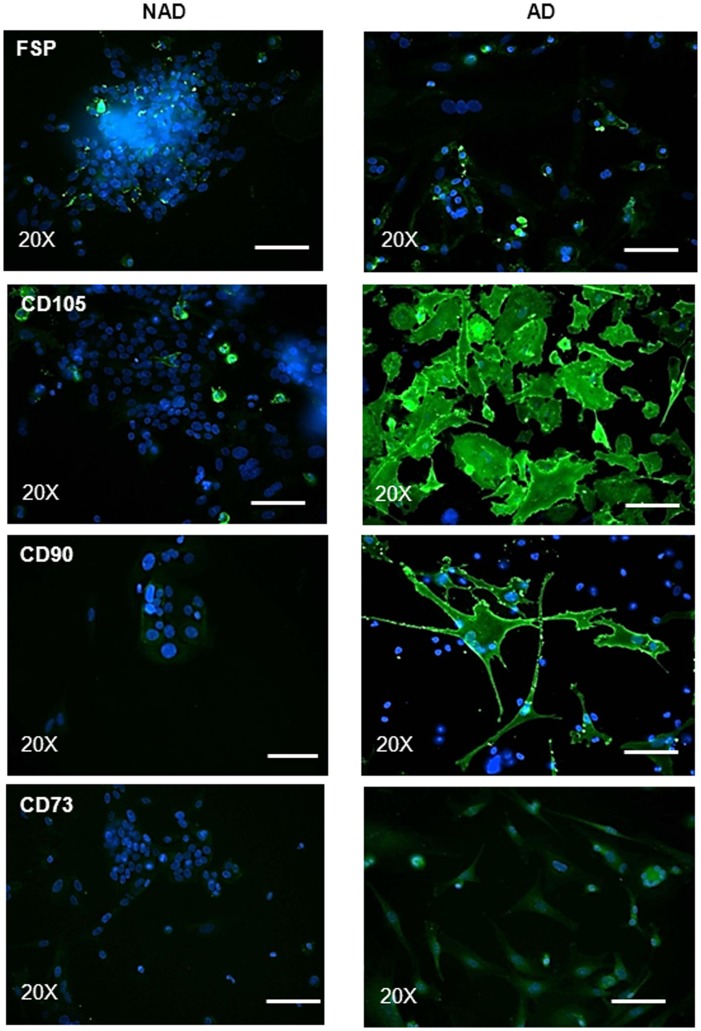
Expression and immunolocalization of MSC markers by immunofluorescence. Immunofluorescence study was performed on purified NAD and AD cells as described in Figure 3. Images are representative of three independent samples. Magnification was 200×; scale bar = 50 µm.

### Patients

#### Human ethics statement

Ascites was collected from patients diagnosed with advanced-stage serous ovarian carcinoma, after obtaining written informed consent under protocols approved by the Human Research and Ethics Committee (HREC # 09/09) of The Royal Women’s Hospital, Melbourne, Australia.

The histopathological diagnosis, including tumor grades and stage were determined by independent staff pathologists as part of the clinical diagnosis (Table 1). Ascites was obtained from patients during surgery with primary carcinoma (CN patients). In other instances, ascites was collected from a group of patients at the time of recurrence (CR patients). These patients had developed recurrent disease within 6–20 months of first line of chemotherapy. Patients in this group were not all treated the same as they had previously received combinations of chemotherapy consisting of paclitaxel, carboplatinum and other drugs such as doxorubicin, gemcitabine, docetaxel, cyclophosphamide and topotecan after each recurrent episode (Table 1)**.** Samples were collected from patients during the treatment regimen as described in Table 1.

**Figure 5:**
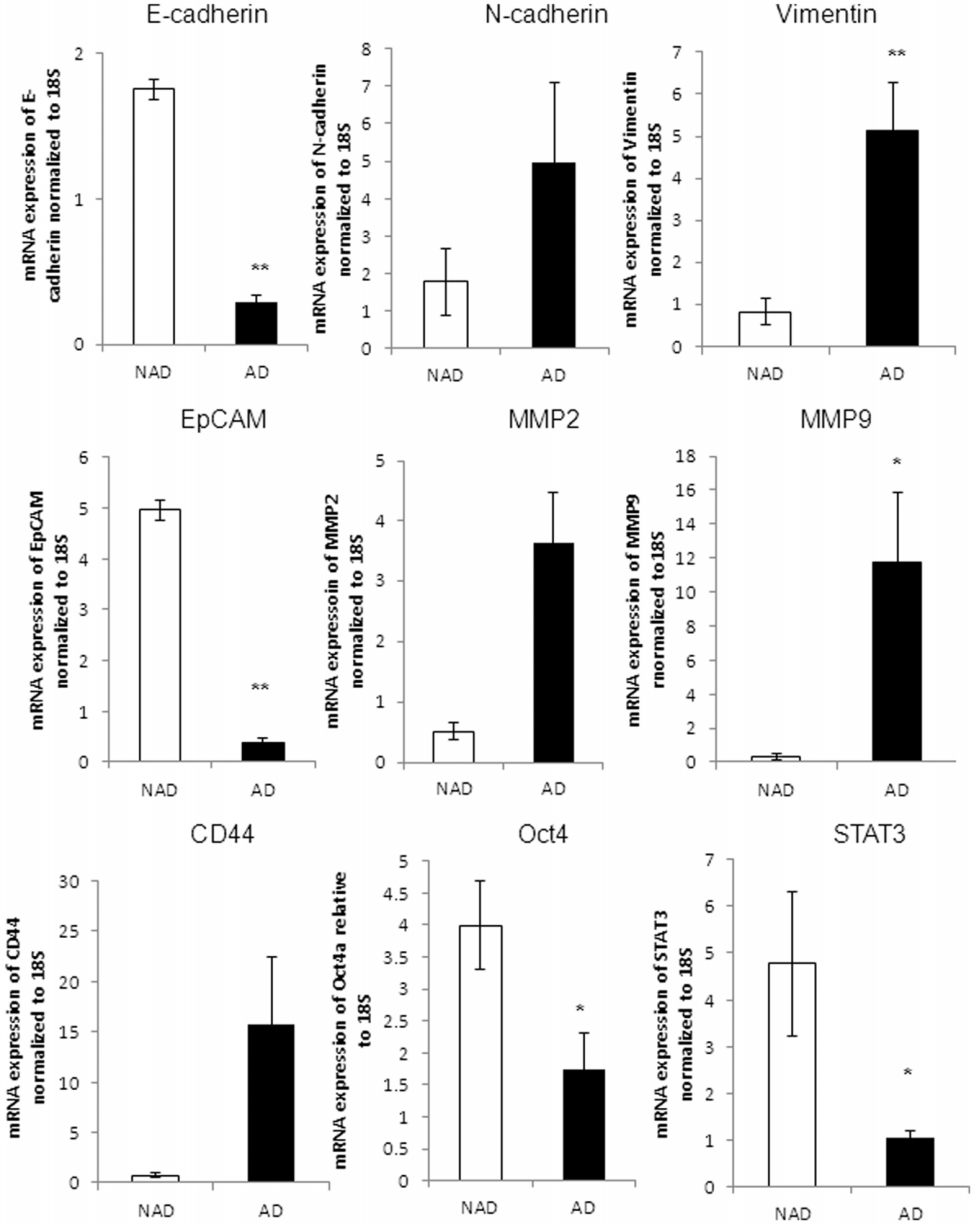
mRNA expression of epithelial, mesenchymal and CSC markers in isolated ascites cells. qPCR was performed on purified NAD and AD populations as described in the Methods and Materials. Yields were converted to femtograms based on the standard curve for each PCR product, and the resultant mRNA levels were normalized to the 18S mRNA level per sample. The data were calculated from the results of eight independent samples assessed in triplicate. Significantly different in AD versus NAD cells *(p<0.05) and **(p<0.01).

### Preparation of Cells from Ascites of Ovarian Cancer Patients

The volume of ascites varied between patients. CN patients had lower ascites volumes (100 ml−2L), compared to CR patients (100 ml−17 L). However, in order to standardise the experimental protocol only 500 ml of ascites was used to collect cells. Contaminating red blood cells in the cell pellet of ascites were removed by hypotonic lysis in sterile MilliQ H_2_O. The bulk of ascites cells were seeded on low attachment plates (Corning Incorporated, NY) in MCDB:DMEM (50∶50) growth medium supplemented with fetal bovine serum (10%), glutamine (2 mM) and penicillin/streptomycin (2 mM) (Life Technologies, CA, USA). Cells were maintained at 37°C in the presence of 5% CO_2_. Under these conditions, NAD cells floated as spheroids in the medium while AD cells attached to low attachment plates. After 2–3 days, floating NAD spheroids (dispersed by pipetting) and AD cells were screened for CA125, EpCAM, cyt 7 and FSP by flow cytometry. AD cells were maintained in plastic tissue culture flasks while NAD spheroids were maintained on low attachment plates. Cells were passaged weekly and experiments were performed within 1–2 passages.

**Figure 6:**
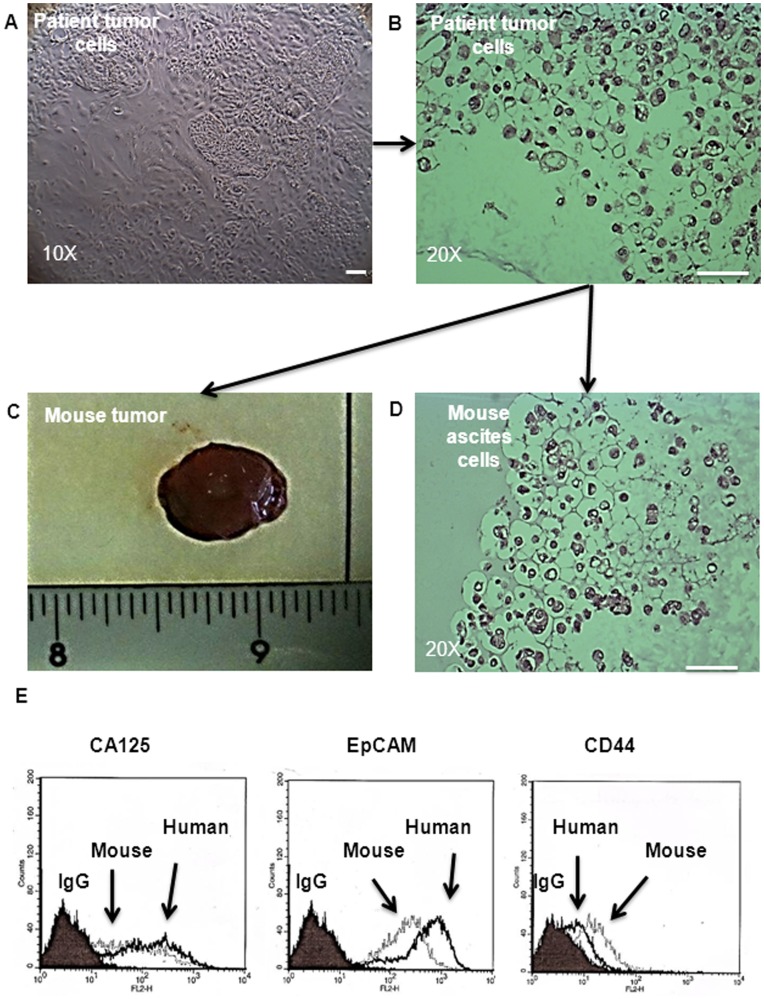
Tumorigenic properties of NAD and AD cells purified from the ascites of CR patients. (**A**) A phase contrast microscope image of NAD cells adhered to plastic before preparing the cell suspension for i.p. injection; (**B**) H and E staining of agarose embedded patient sample before injection; (**C**) image of solid tumor obtained from a mouse fourteen weeks after i.p. injection of NAD cells (5×10^6^); (**D**) H and E staining of mouse ascites NAD cells embedded on agarose; (**E**) Flow cytometric comparison of the expression of CA125, EpCAM and CD44 between the patient’s and mouse ascites cells. Results are representative of two independent samples. The filled histogram in each figure represents control IgG, black lines indicate protein expression in human cells, broken lines indicate the expression of the protein in mouse ascites cells.

### Cell Counts and Proliferation Assay

AD cells and NAD spheroids collected from 1 ml of ascites were allowed to adhere on tissue culture plates for 24 h, following which, cells were trypsinized and counted by Trypan Blue Exclusion method. The number of AD cells, and cells within the NAD spheroids were calculated and the percentage of viable cells in the AD and NAD spheroid populations was evaluated by calculating the total number of cells present in both AD and NAD populations of 1 ml of ascites of each patient.

**Figure 7:**
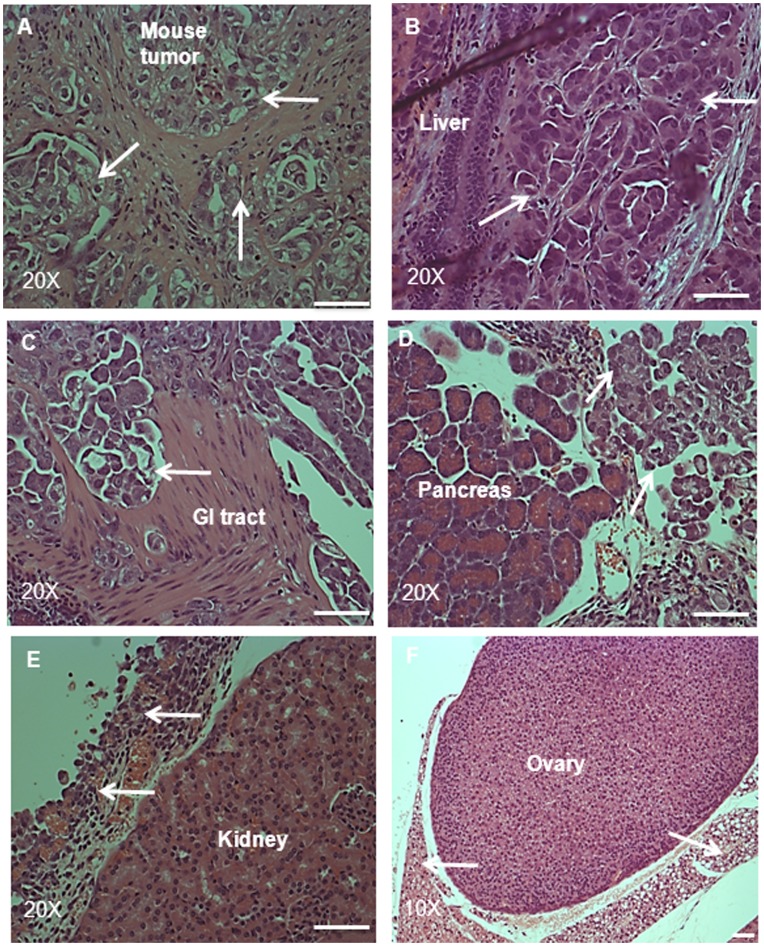
H and E staining of tumor and the associated infiltrated organs in a mouse. Histological images of (**A**) tumor, (**B**) liver, (**C**) GI tract and (**D**) pancreas from a mouse injected i.p. with NAD cells (5×10^6^). Arrows in the tumor (**A**) indicate pockets of tumor cells surrounded by connective tissue. (**B–D**) arrows indicate tumor cells invading the respective organs. H and E staining of (**E**) kidney and (**F**) ovary from a mouse injected with NAD cells (5×10^6^). Arrows indicate tumor cells surrounding the organs without invasion. Magnification was 200× for all, except ovary which had magnification of 100×, scale bar = 50 µm.

^3^[H]-Thymidine uptake assay was performed as described previously [20]. Briefly, 1×10^5^ NAD or AD cells were seeded in triplicate on 24 well plates. After 2, 4 and 6 days, 0.5 µCi of [^3^H] thymidine was added to each well, and cells were incubated at 37°C for an additional 16 h. Cells were washed with PBS, harvested and lysed in 1% Triton and incorporation of [^3^H] thymidine was measured by liquid scintillation counting (Hidex 300SL, LKB Instruments, Australia).

**Figure 8:**
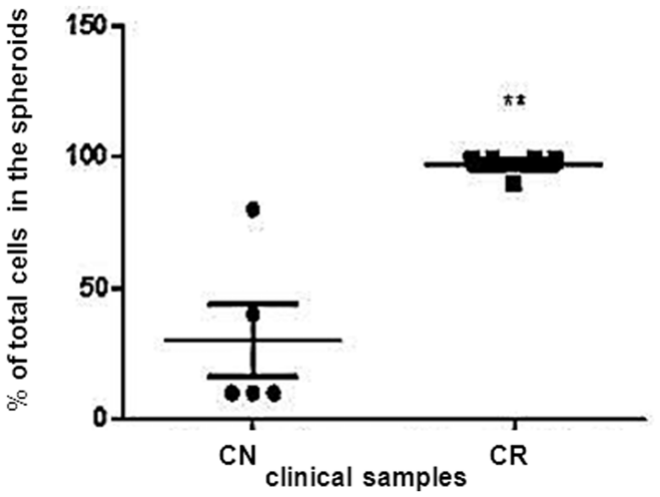
Cellular assessment of NAD and AD cells obtained from CN and CR patients. Percentage distribution of total cells in the NAD and AD populations of ascites of CN (n = 5) and CR (n = 5) patients was determined by Trypan Blue Exclusion assay. Results are mean±SEM of five independent samples assessed in triplicate. Significantly different in CR versus CN samples, **(p<0.01).

For the determination of growth inhibitory concentration (GI50), 1×10^5^ NAD or AD cells were allowed to adhere on 24 well plates for 24 h in triplicate. Both NAD and AD cells were treated with different concentrations of cisplatin (0.5 µg/ml-10 µg/ml) for 48 h before the addition of 0.5 µCi of [^3^H] thymidine for 16 h. The level of [^3^H] thymidine incorporation was determined as described above.

**Figure 9:**
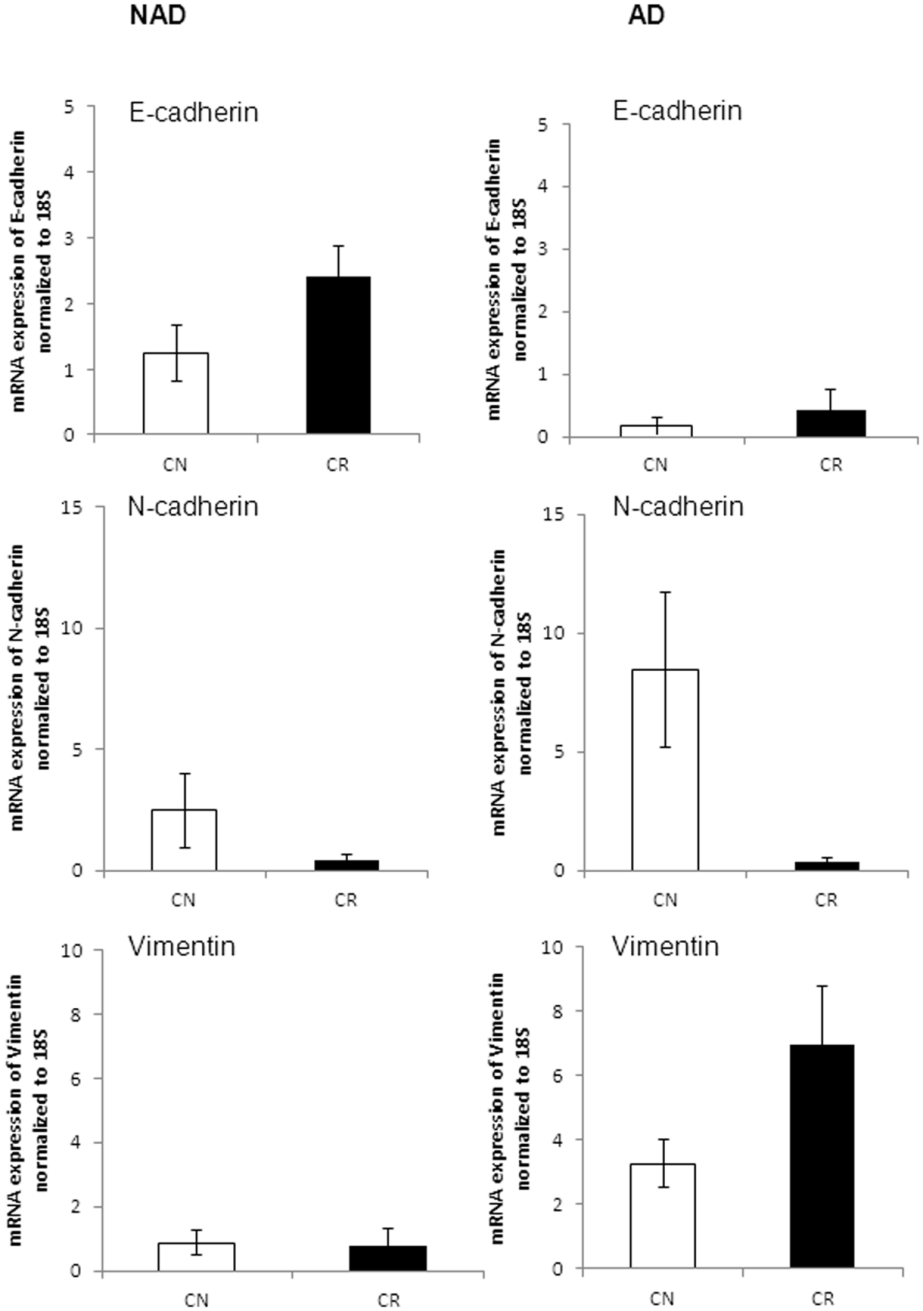
mRNA expression of selective representative epithelial/mesenchymal markers in isolated cells obtained from CN and CR patients. qPCR was performed as described in the Methods and Materials on the purified NAD and AD populations of cells isolated from the ascites of CN (n = 4) and CR (n = 4) ascites samples. Results are expressed as described in Figure 5 for four independent samples assessed in triplicate.

**Figure 10:**
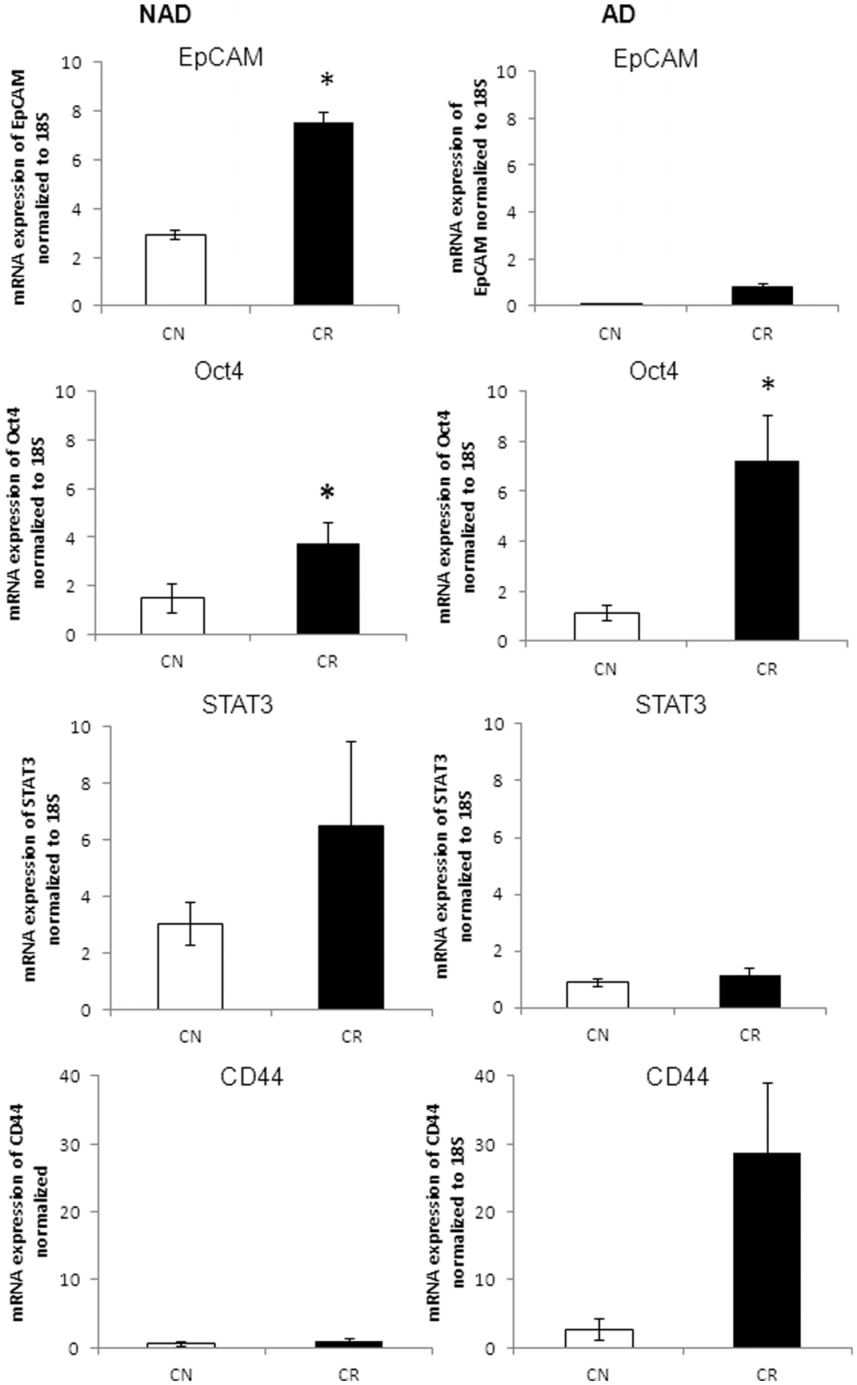
mRNA expression of selective representative CSC markers in purified ascites cells isolated from CN and CR patients. qPCR was performed as described in Figure 9 on the purified NAD and AD populations of cells isolated from CN and CR ascites samples. Results are expressed as described in Figure 9. Significantly different in CR versus CN samples, *(p<0.05).

### Immunofluorescence Analysis

Immunofluorescence analysis was performed as described previously [21]. Images were captured using the Leica TCS SP2 laser, and viewed on a HP workstation using the Leica microsystems TCS SP2 software.

**Figure 11:**
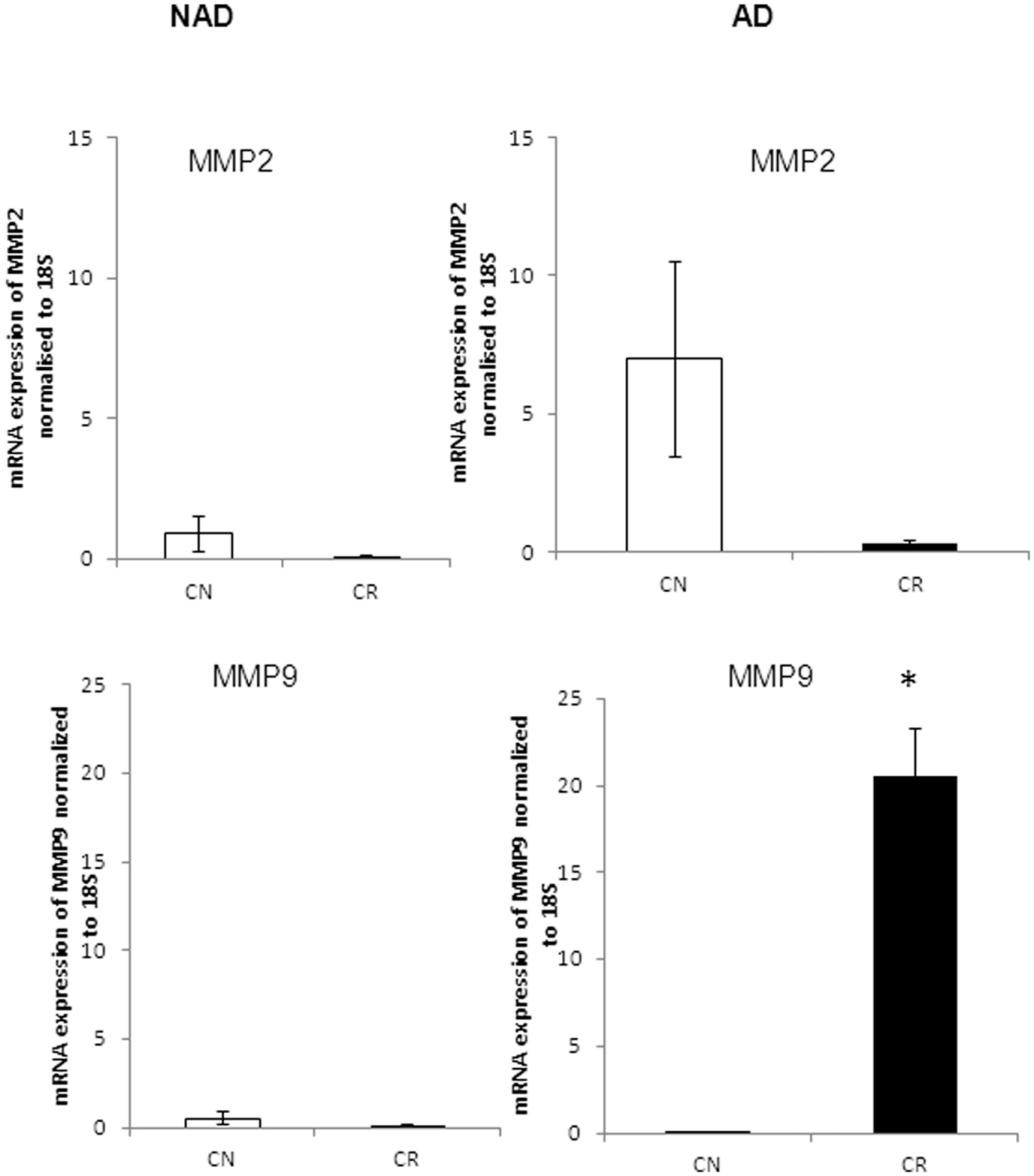
mRNA expression of MMP2 and MMP9 in purified cells from CN and CR patients. qPCR was performed on isolated NAD and AD cells as described in Figure 9. Results are expressed as described in Figure 9. Significantly different in CR versus CN samples, *(p<0.05).

**Figure 12:**
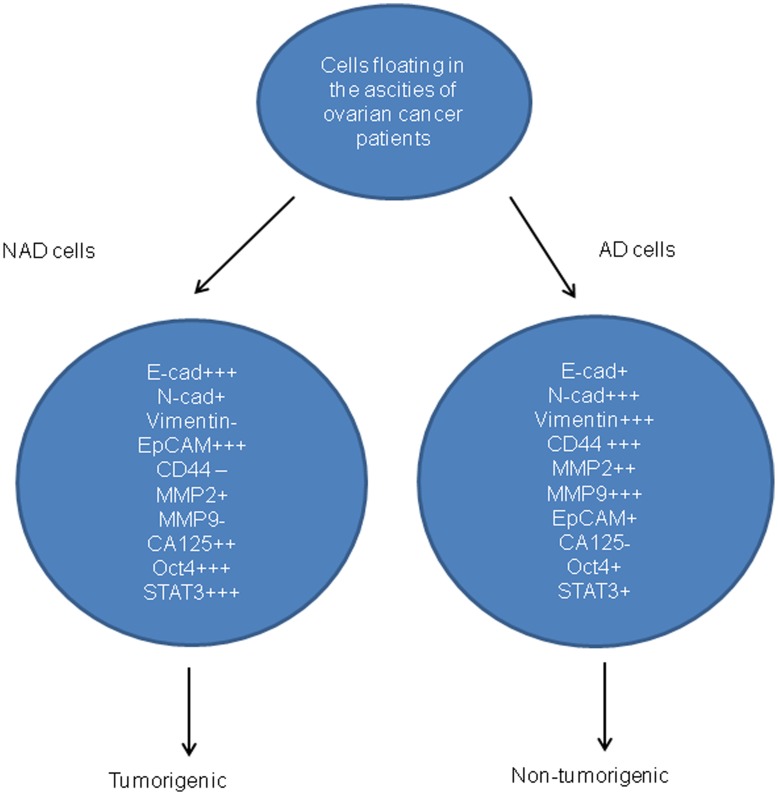
Distribution of markers in isolated cells obtained from the ascites of ovarian cancer patients. The antigens have been scored as high expression (^+++^), moderate expression (^++^), low expression (^+^), and no expression (^−^).

### Flow Cytometry Analysis

The flow cytometry method has been described previously [21]. All data were analysed using Cell Quest software (Becton-Dickinson, Bedford, MA, USA). Results are expressed as mean intensity of fluorescence (MIF).

### RNA Extraction and Quantitative Real Time (q-PCR)

RNA extractions, cDNA synthesis and quantitative determination of mRNA levels of various genes were performed as described previously [21]. Sense and antisense primers were designed against published human sequences for E-cadherin, N-cadherin, Vimentin, Oct4, MMP2, MMP9, EpCAM and CD44. Gel extraction of PCR products was performed using the QiaEX II Agarose gel extraction Kit (Qiagen Australia), as per the manufacturer’s protocol and quantified using the ND-1000 Nanodrop spectrophotometer (NanoDrop Technologies Inc Wilmington, DE, USA). Sequences and products were verified as described previously [22]. Primer pairs used include 5–3′: E cadherin (Entrez Gene ID 999, approved symbol CDH1) forward-GGCACAGATGGTGTGATTACAG; reverse- GTCCCAGGCGTAGACCAAGAAA; N-cadherin (Entrez Gene ID 1000, approved symbol CADH2) forward- AAACAGCAAGCACGGGTTA; reverse- CTTAGGATTGGGGGCAAAAT; vimentin (Entrez Gene ID 7431, approved symbol VIM) forward- CCTACAGGAAGCTGCTGGAA; reverse- GGTCATCGTGATGCTGAGAA; MMP2 (Entrez Gene ID 4313, approved symbol MMP2) forward- AAGGGGATCCAGGAGCTCTA; reverse- GCTTGTCACGTGGTGTCACT; MMP9 (Entrez Gene ID 4318, approved symbol MMP9) forward- TTGACAGCGACAAGAAGTGG; reverse- GCCATTCAC GTCGTCCTTAT; EpCAM (Entrez Gene ID 4072, approved symbol EpCAM) forward- CGTCAATGCCAGTGTACTTCAGTT; reverse- TCCAGTAGGTTCTCACTCGCTCAG; CD44 (Entrez Gene ID 960, approved symbol CD44) forward- CCAATGCCTTTGATGGACCA; reverse- TGTGAGTGTCCATCTGATTC; Oct4 Entrez Gene ID 5460, approved symbol POU5F1): forward- CTCCTGGAGGGCCAGGAATC; reverse- CCACATCGGCCTGTGTATAT; 18S (Entrez Gene ID 100008588, approved symbol RN18S1) forward-GTAACCCGTTGAACCCCATT; reverse-CCATCCAATCGGTAGTAGCG. The mRNA expression of STAT3 was determined using STAT3 probe (Applied Biosystems, Victoria, Australia). Real time PCR was performed using the Applied Biosystems ABI SYBR mix (Victoria, Australia) using Applied Biosystems ABI 7900 HT Fast real-time machine. Yields were converted to fg (femtograms) based on the standard curve for each product and the resulting mRNA levels were normalized to the 18S mRNA level per sample. Each experiment was performed independently a minimum of three times.

**Figure 13:**
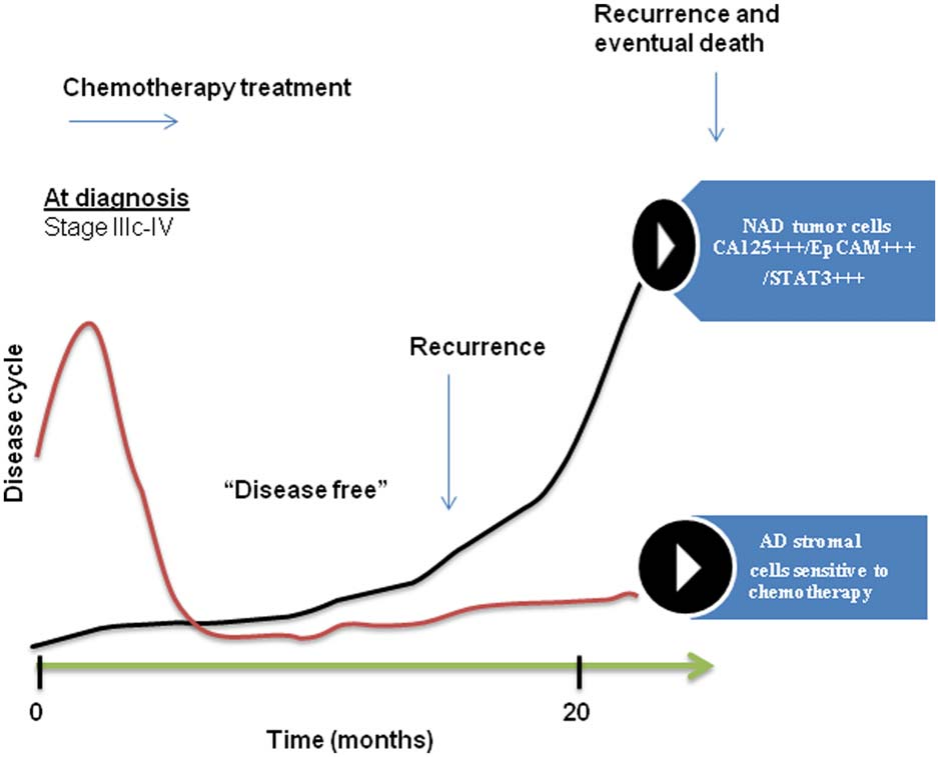
A model of tumor cell progression in the ascites of ovarian cancer patients post-chemotherapy. Most ovarian cancer patients at diagnosis (stage IIIc/IV) present with ascites (CN) which consists of fibroblast-like stromal cells and a very few tumor cells. The majority of these patients (∼80%) after surgery and first line of chemotherapy return with recurrent cancer associated with ascites (CR). During the course of chemotherapy treatment and subsequent relapses, the percentage of stromal cells in the ascites is gradually decreased and the patient with recurrent cancer presents with ascites that consists mostly of CA125^+++^/EpCAM^+++^/STAT3^+++^chemoresistant epithelial NAD tumor cells. These CA125^+++^/EpCAM^+++^/STAT3^+++^ rich tumor cells are the eventual source of extraovarian peritoneal adhesions. These adhesions are the ultimate cause of patient mortality.

### Animal Studies

#### Animal ethics statement

This study was carried out in strict accordance with the recommendations in the Guide for the Care and Use of the Laboratory Animals of the National Health and Medical Research Council of Australia. The experimental protocol was approved by the Ludwig/Department of Surgery, Royal Melbourne Hospital and University of Melbourne’s Animal Ethics Committee (Project-006/11), and was endorsed by the Research and Ethics Committee of Royal Women’s Hospital Melbourne, Australia. Female Balb/c nu/nu mice (age, 6–8 weeks) were obtained from the Animal Resources Centre, Western Australia. Animals were housed in a standard pathogen-free environment with access to food and water.

NAD and AD cells were isolated from the ascites of three CR patients (Table 2). 5×10^6^ cells per group were injected i.p. with a 26-gauge needle into ten mice (six with NAD and four with AD cells). Mice were inspected weekly and tumor progression was monitored based on overall health and body weight until a pre-determined endpoint was reached. Endpoint criteria included loss of body weight exceeding 20% of initial body weight, anorexia, general patterns of diminished well being such as reduced movement and lethargy resulting from lack of interest in daily activities. Mice were euthanized and organs (such as liver, stomach, lungs, gastrointestinal tract, pancreas, uterus, skeletal muscle, colon, kidney, peritoneum, ovaries and spleen), solid tumors and ascites fluid were collected for further examination. Metastatic development was documented by a Royal Women’s Hospital pathologist according to histological examination (H & E staining) of the organs. Ascites tumor cells from mice were maintained on low attachment plates and were embedded on 3% agarose (w/v) for H & E staining. Subsequent characterization of CA125, EpCAM and CD44 expression in cells collected from the ascites of mice was performed as described above.

### Statistical Analysis

Statistical analyses were conducted using GraphPad Prism (Version 5; GraphPad Software Inc., San Diego, CA) and Microsoft Excel. If across the sample set, the means were shown to have a variation at p<0.05 by the F-test then a parametric t-test was conducted for unpaired unequal variances. Data was considered significantly different if p<0.05.

## Results

### Morphology of Cells Collected from Ascites of Cancer Patients

Ascites cells derived from both CN (n = 11) and CR patients (n = 14) were assessed by phase contrast microscopy after seeding on low attachment plates for 24 h. Two distinct populations of cells were observed: (i) multicellular aggregates (spheroids) that floated as three-dimensional structures in the growth medium without attachment (NAD) (Figure 1A), and (ii) spindle-shaped fibroblast-like single cells that adhered to the low attachment plates (AD) (Figure 1 B).

Further morphological assessment of the NAD population revealed numerous three dimensional clusters of loosely compacted spheroids with a central lumen (Figure 1A). There was a considerable variation in the morphology and size of spheroids from different patients as well as within the ascites of the same patient. In some cases, spheroids were in the form of tight balls with a defined outer rim, while others displayed loose aggregates of small cell clusters (Figure 1A). After 24 h culture on tissue culture plastic, most spheroids attached and a clear transformation from a three dimensional structure to flattened cellular clusters containing several layers of adherent cells growing on top of each other was observed (Figure 1C). The periphery of the spheroids exhibited elongated cells moving out of the spheroids, whereas cells towards the centre were more rounded in structure. As the cells moved away from the central core, cell-cell contact was reduced, resulting in the disaggregation of the spheroids (Figure 1C). Alternatively, AD cells attached to the plastic as elongated spindle-like cells and displayed a fibroblast-like morphology (Figure 1D).

### Growth of AD and Spheroid-derived NAD Cells as Monolayer Cultures

The growth pattern of both AD and NAD cells was determined in adherent monolayer cultures by ^3^[H]-thymidine uptake assay. NAD spheroids were dispersed by pipetting and the growth pattern was compared to AD cells. AD cells had nearly 2-fold greater growth response compared to cells dispersed from NAD population (Figure 1E).

### Cisplatin Sensitivity of NAD and AD Cells

The cells from NAD spheroids were dispersed by pipetting and the GI50 value in response to cisplatin was compared to AD cells isolated from the ascites of ovarian cancer patients (n = 3) (Figure 1F). Cells within the NAD spheroids were almost 4-fold more resistant to cisplatin (n = 3, GI50 = 8.8±0.72 µg/ml) compared to AD cells (n = 3, GI50 = 2.4±0.28 µg/ml) (Figure 1 F).

### Assessment of Cell Surface Markers by Flow Cytometry

Cell surface expression of FSP, EpCAM, CA125, CD44 and cyt 7 was determined in AD and NAD cells (dispersed by trypsinization) by flow cytometry. A high level of expression of EpCAM, CA125 and cyt 7 was observed in the cells dispersed from the NAD population, while low/no expression of FSP was detected, and a relatively low level of expression of CD44 was evident in NAD spheroids (Figure 2). On the other hand, AD cells were positive for FSP and CD44, with low/no detectable expression of CA125 and cytokeratin 7 (Figure 2). Low levels of of EpCAM expression were detected in AD cells. This pattern of cell surface marker expression was consistent in all ascites samples (n = 25). No expression of CD34, CD31 and CD45 was observed in either NAD or AD populations by flow cytometry.

### Analysis of CA125, EpCAM, CD44 and Mesenchymal Stem Cell Markers by Immunofluorescence

We next analyzed the expression and localization of CA125, EpCAM, CD44 and mesenchymal stem cell markers in ascites samples by immunofluorescence. Consistent with the flow cytometry results, CA125 was undetected in the AD population, while the cells within the NAD spheroids demonstrated strong three dimensional diffuse expression of CA125 which was prominent in the peripheral membranes of some cells (Figure 3). Very few EpCAM-positive cells were present in the AD population (Figure 3). In contrast, dense peripheral membrane staining for EpCAM was observed in almost all NAD spheroids. Diffuse cytoplasmic staining was present in some spheroids, but the staining was much stronger on the cells lining the periphery of the spheroids (Figure 3). On the other hand, the expression of CD44 was confined to the periphery of the NAD spheroids and was detected in cells that were moving out of spheroids. A strong staining of CD44 was evident on the membrane of almost all AD cells (Figure 3).

As stromal fibroblasts and mesenchymal stem cells (MSC) have been shown to share common properties [23], we assessed the expression of commonly known MSC markers (CD90, CD73 and CD105) in both NAD and AD populations of ascites samples (Figure 4). Scattered diffuse staining of FSP was evident in AD cells (Figure 4). Few FSP-positive cells were observed in NAD spheroids, and these were the cells moving out of the spheroids. Strong expression of CD105 and CD90 was present in AD cells. The expression of these proteins was detected throughout the cytoplasm and at the plasma membrane of the cells. Weak expression of CD73 was present in AD cells. On the other hand, low/no expression of CD90 and CD73 was observed in NAD spheroids. However, CD105 staining was evident in very few scattered cells moving out of the spheroids (Figure 4).

### Quantitative Assessment of Selective Representative Markers at the mRNA Level

To further assess quantitative differences in the expression of epithelial and mesenchymal markers by the NAD and AD populations, we compared the mRNA expression levels of some of the markers by q-PCR. Cells within the NAD spheroids demonstrated a significantly higher expression level of E-cadherin and EpCAM compared to AD cells (p<0.01) (Figure 5). On the other hand, AD cells demonstrated a significantly high mRNA expression level of vimentin and MMP9 compared to cells within the NAD spheroids (p<0.01, p<0.05) (Figure 5). The mean expression of N-cadherin, CD44 and MMP2 appeared higher by 2.5, 7 and 15-fold respectively in the AD population, but no significance was observed (p<0.2, p<0.06, p<0.17). The expression of STAT3 and Oct4 was significantly higher in NAD compared to AD population (p<0.05) (Figure 5).

### Assessment of Tumorigenic Potential of NAD and AD Populations by in vivo Mouse Model

Tumor xenograft experiments were performed to determine the tumorigenic potential of NAD and AD populations isolated from the ascites of three CR patients by i.p. inoculation of 5×10^6^ NAD or AD cells per mouse. Five out of the six mice injected with NAD cells (as a cell suspension) (Figure 6A) developed ascites with solid tumors (<0.5 cm^3^) (n = 3) (Figure 6C) or small lesions (n = 2) in the peritoneum. A tumor latency period of twelve to fourteen weeks after transplantation was observed. Tumors weighing 0.8±0.2 g and ascites (∼0.5 ml) were observed in all five mice that developed tumors (Figure 6C). In contrast, no tumors were observed in mice (n = 4) injected with the same number of AD cells even twenty weeks following i.p. injection.

Mice that developed tumors displayed distended abdomens by nine to eleven weeks suggesting the presence of ascites. Dense, turbid ascites packed with cells were collected from mice that developed tumors. Following seeding of the mouse ascites onto low attachment plates almost all of the cells floated as cellular aggregates (spheroids) and no adherent population was observed. Further H & E assessment of mouse ascites cells showed a similar profile as the donor patient’s spheroid cells (Figures 6B and D). To elucidate whether the tumorigenic and epithelial profiles of the patient’s spheroid cells were retained in the ascites of mice, we assessed the expression of CA125 and EpCAM by flow cytometry. The expression of CA125 remained relatively unchanged in mouse ascites cells compared to that of the donor patients (Figure 6E). However, a slight decrease in the expression of EpCAM and a corresponding increase in the expression of CD44 were observed in mouse ascites cells compared to human donor ascites cells (Figure 6E).

H & E staining of the solid tumors that developed in mice demonstrated pockets of epithelial spheroids surrounded by fibrous tissues (Figure 7A). Representative H & E sections of the organs in the peritoneum of mice that developed tumors demonstrated dissemination of tumor cells in the gastrointestinal tract, liver and pancreas (Figures 7B–D). In the gastrointestinal tract, small pockets of differentiated tumor cells surrounded by fibrous connective tissue were observed, similar to those observed in the solid tumors (Figure 7C). However, in the pancreas and liver, tumor cells disseminated by forming an invading edge which was surrounded by fibrous connective tissue or blood cells (Figures 7B and D). In the ovary and kidney, tumor cells surrounded the organs without obvious invasion (Figures 7E and F). However, no infiltration of tumor cells was observed in the spleen and skeletal muscle (images not shown).

### Cellular Assessment of Selective Representative Markers in CN and CR Ascites Samples

To better understand the composition of ascites obtained from CN and CR patients, we next assessed the proportion of NAD and AD populations in the ascites of CN and CR patients. Ascites from CR patients (n = 5) was predominantly made up of NAD spheroids which when dispersed and counted, constituted an average of ∼95% of total cells (Figure 8). In CN patients (n = 5) the proportion of NAD spheroids varied and constituted an average of 25% of total cells (Figure 8) (p<0.05).

q-PCR was performed to further determine whether this quantitative estimation of NAD spheroid and AD cells in CN and CR ascites samples conforms to the quantitative expression of epithelial and mesenchymal markers observed in both populations. mRNA expression levels of E-cadherin, N-cadherin, vimentin, EpCAM, Oct4, STAT3, CD44, MMP2 and MMP9 were determined in the NAD and AD populations of CN (n = 4) and CR (n = 4) ascites samples. NAD spheroids obtained from CR patients exhibited characteristics indicative of an epithelial nature over spheroids derived from CN patients as demonstrated by a 2-fold enhanced mean expression of E-cadherin (p<0.1), and a corresponding decrease in the expression of N-cadherin in CR compared to CN NAD population (Figure 9). The AD cells of both CN and CR patients had very low expression of E-cadherin, while there was an enhanced mean expression of N-cadherin in the AD population of CN patients (∼16-fold, p<0.08) compared to CR patients (Figure 9). The mean expression of vimentin was 2-fold higher (p<0.1) in the AD cells of CR patients, while no difference in the expression of vimentin was observed in NAD populations of CN and CR patients (Figure 9).

We next analyzed the expression of selective representative CSC markers in the NAD spheroids and AD cells of CN and CR patients. Compared to CN patients, there was a significantly enhanced expression of EpCAM in the NAD spheroids of CR patients (p<0.05) (Figure 10). The expression of Oct4 was significantly enhanced in the NAD and AD populations of CR patients compared to CN patients (p<0.05). The NAD spheroids of CR patients had an enhanced mean expression of STAT3 (2-fold, p<0.2) compared to CN patients (Figure 10). The mean mRNA expression of CD44 in the AD cells of CR patients was approximately 7-fold higher (p<0.08) compared to CN patients (Figure 10).

Compared to CR patients, a high mean expression of MMP2 (p<0.1) was observed in the AD cells of CN patients, while the expression of MMP2 was negligible in NAD cells of both CN and CR patients (Figure 11). Contrary to MMP2 expression, the expression of MMP9 was significantly higher (p<0.05) in the AD cells of CR patients (Figure 11). Very low expression of MMP9 was observed in the NAD cells of CN and CR patients.

## Discussion

In this study, we used a simple method to demonstrate the phenotypic characteristics of two different populations of cells found in the ascites of patients with advanced-stage serous ovarian carcinoma. We report for the first time a distinct separation of ascites cells into NAD epithelial spheroids and AD mesenchymal cells. Unlike cells grown on plastic (which undergo EMT) NAD spheroids (EpCAM^+++^) maintained on low attachment plates preserved their differentiation status with no loss of epithelial characteristics observed during the course of the experiments. This method established to isolate ascites-derived tumor cells from ovarian cancer patients is distinct from other published methods which rely on the use of plastic tissue culture ware [24]. This results in the dedifferentiation of primary human tumor cells to mesenchymal cells with a consequential loss of *in vivo* characteristics. Other methods such as EpCAM-coated [25] or mimetic peptide-based [26] magnetic beads have also been used to isolate tumor cells from ascites, but these methods are not only expensive but time-consuming and result in inconsistent low yields of cells [25].

We demonstrate that spindle-shaped AD cells isolated from ascites have enhanced proliferative capacity and shared antigen profiles in common with stromal fibroblasts (FSP+) and MSC (CD90, CD105, CD73), consistent with recent studies that demonstrated similarities between tumor-associated fibroblasts and MSC [23]. The fact that these cells lacked CA125 expression and had high expression of N-cadherin and vimentin suggests the presence of a non-tumorigenic cell population in ascites. In contrast, the NAD spheroids had high expression of CA125, E-cadherin and EpCAM consistent with the phenotype of epithelial tumorigenic cells [27]–[28]. Low expression of MSC markers was only observed in cells moving out of the spheroids, suggesting the induction of EMT and MSC characteristics in disaggregating cells. Whether this observation is an artifact of culture conditions or is a representation of the *in vivo* situation in patients remains to be established. Notably, sustained incubation of the NAD component in mouse peritoneum for more than 12 weeks did not lead to a detectable AD population *in vivo*.

Tumor-associated MSC have been shown to be derived from circulatory MSC’s or resident tissue stem cells [29]–[30], and have been shown to differentiate into fibroblast-like cells within the tumor stroma [29]. MSC have also been shown to induce EMT and CSC in breast tumor cells [31]–[32], and shown to affect metastasis [33]. A recent study has reported the tumor promoting presence of MSC in ovarian tumors [34]. Whether the MSC in the ascites of ovarian cancer patients have a similar tumor promoting and EMT initiating roles in patients is yet not known, and is the subject of ongoing studies.

Quantitative measurement of epithelial and mesenchymal markers at the mRNA levels indicated that the majority of the epithelial-specific E-cadherin^+++^ and EpCAM^+++^ population resided within the cells of the NAD spheroids while the mesenchymal-specific N-Cadherin^+++^, vimentin^+++^ and CD44^+++^ population dominated the AD population. AD cells had high expression of MMP2 and MMP9, proteases involved with matrix remodeling during tumor dissemination [35]. MMPs secreted by tumor-associated fibroblasts have been shown to play a critical role in ECM degradation during cancer dissemination [35]. In this context, high expression of MMP2 and MMP9 in the fibroblast-like AD mesenchymal population was not unexpected. A schematic diagram of the distribution of these markers in NAD and AD populations is presented in Figure 12.

Recently, the existence of CSCs has been shown to play a significant role in ovarian tumor progression [36], [37]. We report that the NAD epithelial spheroids were rich in endogenous EpCAM^+++^, STAT3^+++^ and Oct4^+++^ expression, the common factors/transcription factors distinguished as CSC markers in recent literature [38]–[39]. These findings are consistent with recent studies that have demonstrated the existence of CSCs in the ascites of ovarian cancer patients [15] [40]. However, a recent study has reported isolation of mesenchymal cells with stem cell-like characteristics derived from the ascites in late-stage and recurrent tumors [24]. The major drawback of this study was that cells were maintained long-term (∼2 months) in adherent cultures in the presence of EMT promoting growth factors such as epidermal growth factor and fibroblast growth factor. Hence, the development of CSC-like characteristics in mesenchymal cells may be due to the initiation of EMT by paracrine factors and not the inherent phenotype of the tumor cells itself.

A xenograft model in which a specific phenotype of human cells isolated from patients can be propagated into tumors provides an opportunity to determine the tumorigenic potential of the specific phenotype of isolated human cells. While subcutaneous mouse xenograft models have been extensively used for the study of cell biology and chemotherapy resistance in solid cancers and even in ovarian cancer [40], their anatomical localization bears little resemblance to the *in vivo* scenario of ovarian cancer patients. The majority of patients with ovarian cancer present with disseminated intra-peritoneal disease, and metastasis outside the peritoneal cavity is a rare event [3]. Hence, i.p. mouse xenografts provide an avenue to determine the pattern of ovarian cancer metastasis to peritoneal organs. With this objective in mind, an i.p. tumor xenograft model was established in which the AD and NAD population of cells isolated from the ascites of three CR patients were injected i.p. in equal numbers into female nude mice. Although AD cells did not produce tumors in mice, the NAD epithelial spheroid cells successfully established xenograft tumors. The pattern of tumor growth and metastasis reflected by the NAD spheroids was similar to that observed in advanced-stage ovarian cancer patients. All tumor-bearing mice developed ascites with cellular aggregates resembling those of the human donor ascites samples. Further examination of the mouse ascites derived cellular aggregates revealed high expression of EpCAM and CA125, consistent with that of the human donor ascites samples. The ability to detect CA125 and EpCAM in the ascites of cellular aggregates of mouse xenografts is reflective of the data that we have observed in the NAD population of ovarian cancer patients. The intra-abdominal spread of NAD spheroid cells to GI tract, pancreas and liver following i.p. inoculation was consistent with what is seen in advanced-stage ovarian cancer patients (stages III/IV) [3]. In mouse xenografts, tumor cells adhered to kidney and ovary but no obvious invasion of tumor into these organs was observed. However, if given extended time, these adherent cells may have developed into micro-metastasis as commonly seen in advanced-stage ovarian cancer patients. Collectively, these findings indicate that the progressive pattern of tumor growth and metastasis in the mouse xenograft model following the i.p. inoculation of human epithelial NAD spheroid cells closely reflected that seen in patients who develop advanced-stage ovarian cancer which accounts for 70% of ovarian cancer patients seen in the clinic [41].

It has been shown that the stage and site specific phenotype of cells within a tumor favor the evolution of a specific phenotype of tumor cells that not only controls tumor progression but also drug resistance [42]. Current studies have also shown that residual cells after chemotherapy treatment secrete soluble factors that provide a favorable microenvironment to facilitate the growth of residual cells [43]. This close relationship between chemotherapy-surviving cells and their secretory microenvironment represent a potential target for cancer therapy. We and others have recently shown the evolution of CSC-like cells in ovarian cancer cell population in response to drug treatment [21] [36]. If such is the case, the presence of CSCs in CR tumors should enhance chemoresistance and correlate with patient prognosis. We demonstrate that mesenchymal AD cells are more susceptible to drug treatment compared to NAD epithelial cells within the spheroids. This may explain the significantly enhanced proportion of epithelial NAD cells in CR patients compared to CN patients. The significant enhanced expression of CSC marker EpCAM on the tumor cells of CR patients is consistent with the epithelial phenotype of that population of cells. EpCAM has been shown to be an independent prognostic marker for reduced survival in ovarian cancer patients [44]. In addition, metastatic and recurrent tumors were found to express significantly higher levels of EpCAM when compared with primary carcinomas [27]. The abundance of EpCAM^+++^epithelial spheroids in CR patients may result from abolishment of stromal components after initial chemotherapy and survival of EpCAM-rich chemotherapy-treated residual cells in an oxygen and growth factor rich ascites microenvironment. In this context, facilitation of rapid proliferation of epithelial cells within the chemotherapy surviving population may serve as a reservoir of increased tumor burden, leading to the eventual mortality in recurrent patients [45]. A conceptual model based on our findings of ovarian tumor progression in cancer patients is depicted in Figure 13.

Recent literature suggests that tumors rich in activated stromal cells perpetuate aggressive malignancies [46]. In that scenario, the reduced stromal component in CR ovarian tumors is consistent with the non-aggressive nature of ovarian cancer where tumor growth is localized within the peritoneum microenvironment and is more dependent on dissemination by adhesion rather than aggressive invasion through the vasculature. Considering that CR patients lack, or are low in stromal component, enhanced expression of CD44 in the stroma of CR patients is intriguing. As CD44 is involved with the assembly of extracellular matrix through the regulation of hylauronan, integrins and tetraspanin family of adhesion molecules [47], one would expect that the expression of CD44 would be high in CN tumors with a greater proportion of stromal component. The tumor promoting as well as tumor initiating potential of CD44 has been observed in many cancers [47]–[48]. High expression of CD44 in the stroma of CR tumors may serve to facilitate either of these functions. Another interesting finding in this study is the distinct profile of MMP2 and MMP9 in CN versus CR patients. Although difficult to explain, a recent study has reported pro-metastatic effects induced by MMP9 in tumor cells and bone marrow derived cells in mouse xenografts after exposure to certain chemotherapies (taxane-based) [49]. The expression of MMP2 in that study however remained unchanged.

Our observations in ovarian cancer that the more epithelial (EpCAM-rich) NAD spheroids exhibit the enhanced malignant potential in mice and demonstrate an increasing trend of mRNA expression of selective CSC-like markers, are not entirely consistent with the relationship that has been developed between EMT and CSC in mammary cancer [8]–[9]. However, recent analysis of EpCAM-low and –high subcomponents in predominantly basal human mammary cell lines has partitioned certain aspects of stem-like activity (differentialtion, morphogenic potential, high drug efflux potential) to the EpCAM high population resembling luminal progenitor cells, while the EpCAM-low cells exhibit EMT, invasiveness and mammosphere formation associated with CSC [50]. This is consistent with the emerging association between an altered luminal progenitor profile in basal breast cancers associated with BRCA1 mutations, rather than the mesenchymal profile seen in breast CSC. Indeed, association between increased pluripotency and epithelial subcomponent of human bladder and prostatic carcinoma cells has recently been demonstrated [13]. Thus, the relationship between malignant potential, CSC nature and epithelial mesenchymal plasticity in different cancer types is an evolving field.

The main finding of this study is the demonstration of a distinct phenotype of tumor cells (CA125^+++^EpCAM^+++^/STAT3^+++^), as well as stromal cells (CD44^+++^/Oct4^+++^) isolated from the ascites of a limited number of CR patients (n = 4). Using a novel purification method we demonstrate for the first time that tumor cells isolated from the ascites of recurrent ovarian cancer patients are enriched with EPCAM^+++^/STAT3^+++^ tumor cells compared to cells isolated from the ascites of CN patients. Hence, targeting EpCAM^+++^ ovarian tumor cells and/or the associated STAT3 pathway in combination with chemotherapy may help achieve durable clinical responses in recurrent ovarian cancer patients. In this context, a recent study has demonstrated that targeting STAT3 with a small molecule inhibitor can suppress ovarian cancer growth and potentiate the effect of cisplatin in a mouse xenograft model [51]. Future studies that elaborate on these findings in preclinical and clinical settings are needed to develop strategies for the better management of recurrent ovarian cancer patients.
